# The risk of rash for cancer patients treated with PD-1/PD-L1 inhibitors: An updated systematic review and meta-analysis

**DOI:** 10.1097/MD.0000000000049720

**Published:** 2026-07-10

**Authors:** Qunqun Jiang, Junru Liu, Zhuoqi Li, Chi Zhang, Qi Dang, Yuan Tian

**Affiliations:** a Dermatological Department, The 970th Hospital of the Joint Logistics Support Force of the People’s Liberation Army of China, Weihai, Shandong, P.R. China; b Department of Dermatology, Yantai Yuhuangding Hospital, Yantai, Shandong, P.R. China; c Department of Radiotherapy Oncology, Affiliated Hospital of Shandong University of Traditional Chinese Medicine, Jinan, China; d Department of Cardiology, The Second Hospital, Cheeloo College of Medicine, Shandong University, Jinan, Shandong, P.R. China; e Phase I Clinical Trial Center, Shandong Cancer Hospital and Institute, Shandong First Medical University, and Shandong Academy of Medical Sciences, Jinan, Shandong, P.R. China.

**Keywords:** cancer, meta-analysis, PD-1, PD-L1, rash, risk

## Abstract

**Background:**

This study was designed to assess the risk of programmed cell death-1 (PD-1) or programmed cell death ligand 1 (PD-L1) related rash in various situations.

**Methods:**

Guided by Preferred Reporting Items for Systematic Reviews and Meta-Analyses, data on rash in clinical trials related to PD-1 or PD-L1 were collected to comprehensively evaluate its incidence risk in various situations.

**Results:**

Ninety-five clinical trials, divided into 12 groups according to treatment regimens, were enrolled for the final comprehensive assessments and analyses. Compared with chemotherapy or placebo alone, both PD-1 and PD-L1 inhibitors increased the risk of developing rashes for all grades (odds ratio [OR] = 1.61, *P* = .0003; OR = 2.94, *P* < .00001). Whether used in combination with chemotherapy or other types of immunosuppressants, similar risk trends were observed (OR = 2.42, *P* < .00001; OR = 2.11, *P* = .0007; OR = 4.49, *P* < .00001; OR = 5.15, *P* = .0006; OR = 3.16, *P* = .0002).

**Conclusion:**

Both PD-1 and PD-L1 increased the risk of rash occurrence, especially when used in combination with other immunosuppressive antitumor drugs.

## 1. Introduction

Immunosuppressive drugs such as programmed cell death-1 (PD-1) and programmed cell death ligand 1 (PD-L1) have shown encouraging antitumor therapeutic effects in many solid tumors.^[1–95]^ With the emergence of various combination treatment regimens based on immunosuppressants, judging treatment side effects has become increasingly difficult.^[1–95]^ Furthermore, a large amount of clinical trial data related to PD-1 or PD-L1 are updated and reported every year, which forces us to reassess the risk of drug side effects.^[1–96]^

Rash, considered a type of skin toxicity, has been frequently reported in several PD-1 or PD-L1 related clinical trials,^[1–95]^ especially for the PD-1 inhibitor Camrelizumab.^[60–63]^ Correlations between drug side effects and clinical efficacy have been frequently discussed,^[97–99]^, while few reports on the clinical efficacy correlation between rash and PD-1/PD-L1 have been found.^[1–96]^ T cell activation, cytokine release, and skin immune cell infiltration are promoted by immunosuppressants, subsequently inducing a rash.^[100–102]^ The appearance of rash would affect the patient’s quality of life and mental states, and when it becomes severe, it might even lead to interruption of treatment. Considering the above factors, we designed this study to update the previous meta-analysis and reevaluate the risk of rash in various complex situations.^[96]^

## 2. Methods

The entire analysis process was implemented according to the Preferred Reporting Items for Systematic Reviews and Meta-Analyses guidelines.^[96,103]^

### 2.1. Qualification certification for all enrolled clinical trials

Phase III clinical trials with completed reporting data should be given priority consideration, while other phases should be considered as alternatives. For the repeatedly reported clinical trials, only the best one could be enrolled in the final comprehensive analysis. Lymphoma and malignant hematoproliferative diseases were excluded. Only data for all grades of rashes were collected. Groups that included at least 3 sets of data were used in the final meta-analysis.

### 2.2. Search strategy for all enrolled clinical trials

All searches were completed using the PubMed website. The language of publication was restricted to English. Phase III randomized clinical trials were considered as priority options; other phases were considered as alternatives, while real-world studies with small sample sizes were not taken into account. Based on the reference to participants, interventions, comparisons, outcomes, the search strategy would be adjusted appropriately to retrieve as much clinical trial data as possible.^[101]^ Words related to solid tumors were used as keywords for searching, without limiting specific tumor types. The types of immune drugs, including PD-1, PD-L1, CTLA-4, and their corresponding specific drug names or codes, were also used for searching, and then intersected with the previous search results. The time frame involved was limited to the past 10 years, while some reliable and truthful reporting data obtained from the published literature might be moderately adjusted.

### 2.3. Assessment of potential bias

All clinical trial data were required to undergo quality evaluation before inclusion in the final analysis. Random sequence generation (selection bias), allocation concealment (selection bias), blinding of participants and personnel (performance bias), blinding of outcome assessment (detection bias), incomplete outcome data (attrition bias), selective reporting (reporting bias), and other biases were first considered for all clinical trials and completed using the Newcastle–Ottawa scale.^[104]^ Egger test was adopted for evaluation of publication and presented in the form of funnel plots.^[105]^ As usual, *P* < .05 was deemed statistically significant.

### 2.4. Analyses results reports

The basic characteristics of all enrolled clinical trials, including the first author, trial name, NCT number, year, treatment regimen, phase, previous therapy, rash across all grades, and participants were collected and displayed in a single table. The analysis results were mainly based on the risk assessment of rash across all grades. The classification methods of subgroup analysis were mainly determined by the heterogeneity of the analysis results, types of immunosuppressants involved, and different treatment regimens. Whether the results of the subgroup analysis should be presented separately needs to be discussed based on specific circumstances.

### 2.5. Heterogeneity and statistical analyses

Heterogeneity assessments were completed using Cochrane *Q* and *I*^2^ statistics, as suggested by Higgins and colleagues.^[103,104]^ Publication bias evaluation mainly referred to the results of the Harbor test.^[104,105]^ Two separation thresholds (25% and 50%) of *I*^2^ were used to classify heterogeneity into 3 levels: low (≤25%), medium (25–50%), and high (≥50%).^[106,107]^ All analyses were performed using Review Manager version 5.3. Odds ratios (OR) and 95% confidence intervals (CI), calculated using the random effect model, were considered.^[108]^ The fixed effect model was used only for the construction of the funnel plots. All statistical tests were bilateral, and statistical significance was set at *P* < .05.

## 3. Results

### 3.1. Basic characteristics for all enrolled clinical trials

**Figure 1.**
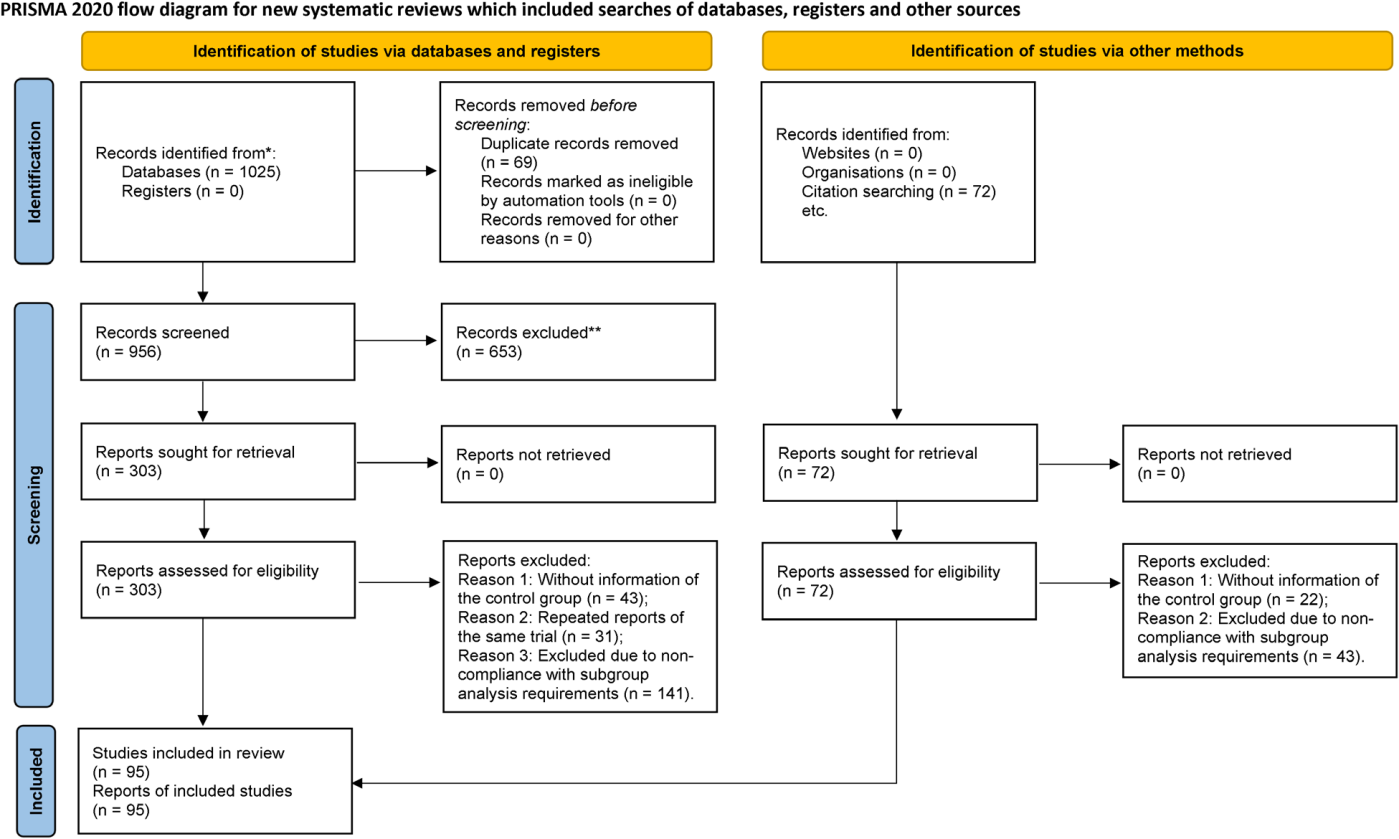
The flow diagram of clinical trial screening for this study.

After screening thousands of studies, data from 95 clinical trials were extracted and used for the final comprehensive analysis (Fig. 1).^[1–95]^ The basic characteristics of all enrolled clinical trials are summarized in Table 1,^[1–95]^ and quality assessments were completed and are provided in Figure S1, Supplemental Digital Content 1. For the same clinical trial that had been repeatedly reported multiple times, only the most comprehensive results were retained (Table 1).

**Table 1:** Basic characteristic information of all collected clinical trial.

No.	Reference	NCT number	Drug	Treatment regimens	Patients	Rash	Previous therapy	RCT	Phase	Tumor type
		PD-1/PD-L1 vs chemotherapy								
1	Borghaei H, et al^[1]^	NCT01673867 (CheckMate 057)	Nivolumab (PD-1)	Nivolumab vs Docetaxel	555	49	Yes	Yes	III	Advanced non-squamous NSCLC
2	Weber JS, et al^[2]^	NCT01721746 (CheckMate 037)	Nivolumab (PD-1)	Nivolumab vs (Dacarbazine/Paclitaxel + Carboplatin)	370	30	No	Yes	III	Advanced melanoma
3	Brahmer J, et al^[3]^	NCT01642004 (CheckMate 017)	Nivolumab (PD-1)	Nivolumab vs Docetaxel	260	13	Yes	Yes	III	Advanced squamous cell NSCLC
4	Herbst RS, et al A^[4]^	NCT01905657 (KEYNOTE-010)	Pembrolizumab (PD-1)	(Pembrolizumab 2 mg/kg) vs (Pembrolizumab 10 mg/kg)	991	73	Yes		II/III	Advanced NSCLC
	Herbst RS, et al B^[4]^			(Pembrolizumab 2 mg/kg) vs Docetaxel		43		Yes		
	Herbst RS, et al C^[4]^			(Pembrolizumab 10 mg/kg) vs Docetaxel		58				
5	Bellmunt J, et al^[5]^	KEYNOTE-045 (NCT02256436)	Pembrolizumab (PD-1)	Pembrolizumab vs chemotherapy	321	45	Yes	Yes	III	Urothelial carcinoma
6	Hida T, et al A^[6]^	OAK	Atezolizumab (PD-L1)	Atezolizumab vs Docetaxel	1086	88	Yes	Yes	III	NSCLC
	Hida T, et al B^[6]^			Atezolizumab vs Docetaxel (Japanese)	101	22	Yes	Yes	III	NSCLC
7	Hellmann MD, et al A^[7]^	NCT02477826 (CheckMate 227)		(Nivolumab + Ipilimumab) vs Nivolumab	1537	139	No		III	Stage IV or recurrent NSCLC
	Hellmann MD, et al B^[7]^		Nivolumab (PD-1)	(Nivolumab + Ipilimumab) vs chemotherapy		125		Yes		
	Hellmann MD, et al C^[7]^			Nivolumab vs chemotherapy		72				
8	Powles T, et al^[8]^	NCT02302807 (IMvigor211)	Atezolizumab (PD-L1)	Atezolizumab vs chemotherapy (vinflunine paclitaxel or docetaxel)	902	61	Yes	Yes	III	Locally advanced or metastatic UC
9	Wu YL, et al^[9]^	NCT02613507 (CheckMate 078)	Nivolumab (PD-1)	Nivolumab vs Docetaxel	493	43	Yes	Yes	III	Advanced NSCLC
10	Kato K, et al^[10]^	ATTRACTION-3 (NCT02569242)	Nivolumab (PD-1)	Nivolumab vs chemotherapy	417	54	Yes	Yes	III	ESCC
11	Wu YL, et al^[11]^	KEYNOTE-042 China Study	Pembrolizumab (PD-1)	Pembrolizumab vs chemotherapy	263	22	No	Yes	III	NSCLC
12	Hamanishi J, et al^[12]^	NINJA	Nivolumab (PD-1)	Nivolumab vs (GEM or PLD)	311	26	Yes	Yes	III	Ovarian cancer
13	Winer EP, et al^[13]^	NCT02555657 (KEYNOTE-119)	Pembrolizumab (PD-1)	Pembrolizumab vs (Single-drug chemotherapy)	601	8	Yes	Yes	III	Metastatic TNBC
14	Powles T, et al A^[14]^	KEYNOTE-361 (NCT02853305)	Pembrolizumab (PD-1)	Pembrolizumab vs chemotherapy	648	64	No	Yes	III	Advanced UC
15	Pujade-Lauraine E, et al C^[15]^	JAVELIN Ovarian 200	PD-L1	Avelumab vs PLD	364	25	No	Yes	III	Platinum-refractory OC
16	Sezer A, et al^[16]^	NCT03088540 (EMPOWER-Lung 1)	Cemiplimab (PD-1)	Cemiplimab vs chemotherapy	697	26	No	Yes	III	Advanced NSCLC
17	Xu J et al^[17]^	ORIENT-2 (NCT03116152)	Sintilimab (PD-1)	Sintilimab vs chemotherapy	181	4	Yes	Yes	II	ESCC
18	Chan ATC et al^[18]^	KEYNOTE-122 (NCT02611960)	Pembrolizumab (PD-1)	Pembrolizumab vs chemotherapy	228	30	Yes	Yes	III	Metastatic NC
19	Shitara K, et al B^[19]^	KEYNOTE-062 (NCT02494583)	Pembrolizumab (PD-1)	Pembrolizumab vs chemotherapy	499	26	No	Yes	III	Advanced GC
20	Mok TSK, et al^[20]^	NCT02220894 (KEYNOTE-042)	Pembrolizumab (PD-1)	Pembrolizumab vs chemotherapy	1251	73	Yes	Yes	III	Metastatic NSCLC
21	Jassem J, et al^[21]^	IMpower110 (NCT02409342)	Atezolizumab (PD-L1)	Atezolizumab vs chemotherapy	549	73	No	Yes	III	NSCLC
22	Moehler M, et al^[22]^	JAVELIN Gastric 100 (NCT02625610)	Avelumab (PD-L1)	Avelumab vs chemotherapy	499	5	No	Yes	III	GC/GEJC
23	Galsky MD, et al C^[23]^	IMvigor130	Atezolizumab (PD-L1)	Atezolizumab vs chemotherapy	744	119	No	Yes	III	NSCLC
		(PD-1/PD-L1 + chemotherapy) vs chemotherapy						Yes		
24	Gadgeel S, et al^[24]^	KEYNOTE-189 (NCT02578680)	Pembrolizumab (PD-1)	(Pembrolizumab + Pemetrexed + Platinum) vs (Pemetrexed + Platinum)	607	105	No	Yes	III	NSCLC
25	Langer CJ, et al^[25]^	KEYNOTE-021 (NCT02039674)	Pembrolizumab (PD-1)	(Pembrolizumab + Carboplatin + Pemetrexed) vs (Carboplatin + Pemetrexed)	121	25	No	Yes	II	Advanced non-squamous NSCLC
26	Novello S, et al^[26]^	KEYNOTE-407 (NCT02775435)	Pembrolizumab (PD-1)	(Pembrolizumab + chemotherapy) vs chemotherapy	558	84	No	Yes	III	Squamous NSCLC
27	Lu S, et al^[27]^	RATIONALE 304 (NCT03663205)	Tislelizumab (PD-1)	(Tislelizumab + Platinum + Pemetrexed) vs (Platinum + Pemetrexed)	332	60	No	Yes	III	Non-squamous NSCLC
28	Zhou C^[28]^	ORIENT-12 (NCT03629925)	Sintilimab (PD-1)	(Sintilimab + GP) vs GP	357	68	No	Yes	III	NSCLC
29	Forde PM, et al^[29]^	CheckMate 816 (NCT02998528)	Nivolumab (PD-1)	(Nivolumab + chemotherapy) vs chemotherapy	352	16	No	Yes	III	Resectable NSCLC
30	Garassino MC, et al^[30]^	KEYNOTE-189 (NCT02578680)	Pembrolizumab (PD-1)	(Pembrolizumab + chemotherapy) vs chemotherapy	607	116	No	Yes	III	Non-squamous NSCLC
31	Makharadze T, et al^[31]^	EMPOWER-Lung 3 (NCT03409614)	Cemiplimab (PD-1)	(Cemiplimab + chemotherapy) vs chemotherapy	465	21	No	Yes	II	Advanced NSCLC
32	Wang Z, et al^[32]^	CHOICE-01 (NCT03856411)	Toripalimab (PD-1)	(Toripalimab + chemotherapy) vs chemotherapy	464	64	No	Yes	III	Advanced NSCLC
33	West H, et al^[33]^	IMpower130 (NCT02367781)	Atezolizumab (PD-L1)	(Atezolizumab + chemotherapy) vs chemotherapy	705	82	No	Yes	III	Non-squamous NSCLC
34	Zhou C, et al^[34]^	GEMSTONE-302 (NCT03789604)	Sugemalima (PD-L1)	(Sugemalima + Carboplatin + Paclitaxel) vs (Carboplatin + Paclitaxel)	479	62	No	Yes	III	Metastatic NSCLC
35	Johnson ML, et al B^[35]^	POSEIDON (NCT03164616)	Durvalumab (PD-L1)	(Durvalumab + chemotherapy) vs chemotherapy	667	49	No	Yes	III	Metastatic NSCLC
36	Jotte R, et al^[36]^	IMpower131 (NCT02367794)	Atezolizumab (PD-L1)	(Atezolizumab + Carboplatin + Nab-paclitaxel) vs (Carboplatin + Nab-paclitaxel)	668	38	Yes	Yes	III	NSCLC
37	Goldman JW, et al A^[37]^	CASPIAN (NCT03043872)	Durvalumab (PD-L1)	(Durvalumab + EP) vs EP	531	26	No	Yes	III	Ex-SCLC
38	Liu SV, et al^[38]^	IMpower133 (NCT02763579)	Atezolizumab (PD-L1)	(Atezolizumab + CP/ET) vs CP/ET	394	61	No	Yes	I/III	Ex-SCLC
39	Schmid P, et al^[39]^	IMpassion130 (NCT02425891)	Atezolizumab (PD-L1)	(Atezolizumab + Nab-paclitaxel) vs (placebo + Nab-paclitaxel)	890	113	No	Yes	III	TNBC
40	Mittendorf EA, et al^[40]^	IMpassion031 (NCT03197935)	Atezolizumab (PD-L1)	(Atezolizumab + chemotherapy) vs chemotherapy	331	88	No	Yes	III	TNBC
41	Miles D, et al^[41]^	IMpassion131 (NCT03125902)	Atezolizumab (PD-L1)	(Atezolizumab + Paclitaxel) vs (Placebo + Paclitaxel)	649	207	No	Yes	III	Advanced/metastatic TNBC
42	Røssevold AH, et al^[42]^	ALICE (NCT03164993)	Atezolizumab (PD-L1)	(Atezolizumab + chemotherapy) vs chemotherapy	68	37	No	Yes	III	TNBC
43	Schmid P, et al^[43]^	KEYNOTE-522 (NCT03036488)	Pembrolizumab (PD-1)	(Pembrolizumab + chemotherapy) vs chemotherapy	1172	262	No	Yes	III	TNBC
44	Rudin CM, et al^[44]^	KEYNOTE-604 (NCT03066778)	Pembrolizumab (PD-1)	(Pembrolizumab + EP) vs EP	446	43	No	Yes	III	Ex-SCLC
45	Sun JM, et al^[45]^	KEYNOTE-590 (NCT03189719)	Pembrolizumab (PD-1)	(Pembrolizumab + chemotherapy). vs chemotherapy	740	47	No	Yes	III	Advanced ESCC
46	Doki Y, et al A^[46]^	CheckMate 648 (NCT03143153)	Nivolumab (PD-1)	(Nivolumab + chemotherapy) vs chemotherapy	614	29	No	Yes	III	Advanced ESCC
47	Wang ZX, et al^[47]^	JUPITER-06 (NCT03829969)	Toripalimab (PD-1)	(Toripalimab + TP) vs TP	514	83	No	Yes	III	Advanced ESCC
48	Kato K, et al A^[48]^	CheckMate 648/ONO‑4538‑50	Nivolumab (PD-1)	(Nivolumab + chemotherapy) vs chemotherapy	256	8	No	Yes	III	Advanced ESCC
49	Xu J, et al^[49]^	RATIONALE-306 (NCT03783442)	Tislelizumab (PD-1)	(Tislelizumab + chemotherapy) vs chemotherapy	645	47	No	Yes	III	Advanced ESCC
50	Lu Z, et al^[50]^	ORIENT-15 (NCT03748134)	Sintilimab (PD-1)	(Sintilimab + chemotherapy) vs chemotherapy	659	64	No	Yes	III	Advanced ESCC
51	Powles T, et al B^[14]^	KEYNOTE-361 (NCT02853305)	Pembrolizumab (PD-1)	(Pembrolizumab + chemotherapy) vs chemotherapy	993	147	No	Yes	III	UC
	Powles T, et al C^[14]^			(Pembrolizumab + chemotherapy) vs chemotherapy						
52	Galsky MD, et al B^[23]^	IMvigor130 (NCT02807636)	Atezolizumab (PD-L1)	(Atezolizumab + chemotherapy) vs chemotherapy	843	211	No	Yes	III	UC
53	Monk BJ, et al A^[51]^	JAVELIN Ovarian 100 (NCT02718417)	Avelumab (PD-L1)	(Avelumab + chemotherapy) vs chemotherapy	663	91	No	Yes	III	OC
54	Shitara K, et al C^[19]^	KEYNOTE-062 (NCT02494583)	Pembrolizumab (PD-1)	(Pembrolizumab + chemotherapy) vs chemotherapy	494	37	No	Yes	III	GC
55	Xu J^[52]^	ORIENT-16 (NCT03745170)	Sintilimab (PD-1)	(Sintilimab + chemotherapy) vs chemotherapy	648	19	No	Yes	III	GEJC
56	Kang YK, et al^[53]^	ATTRACTION-4 (NCT02746796)	Nivolumab (PD-1)	(Nivolumab + chemotherapy) vs chemotherapy	717	59	No	Yes	III	GC/GEJC
57	Shitara K, et al A^[54]^	CheckMate 649 (NCT02872116)	Nivolumab (PD-1)	(Nivolumab + chemotherapy) vs chemotherapy	1549	89	No	Yes	III	GEJC
58	Colombo N, et al B^[55]^	KEYNOTE-826 (NCT03635567)	Pembrolizumab (PD-1)	(Pembrolizumab + chemotherapy) vs chemotherapy	227	17	No	Yes	III	Cervical cancer
59	Eskander RN, et al A^[56]^	NRG-GY018 (NCT03914612)	Pembrolizumab (PD-1)	(Pembrolizumab + chemotherapy) vs chemotherapy (dMMR)	215	20	No	Yes	III	Advanced endometrial cancer
	Eskander RN, et al B^[56]^			(Pembrolizumab + chemotherapy) vs chemotherapy (pMMR)	550	84				
60	Yang Y, et al^[57]^	RATIONALE-309 (NCT03924986)	Tislelizumab (PD-1)	(Tislelizumab + chemotherapy) vs chemotherapy	263	63	NO	Yes	III	NC
61	Mai HQ, et al^[58]^	NCT03581786	Toripalimab (PD-1)	(Toripalimab + GP) vs GP	289	71	No	Yes	III	NC
62	Huober J, et al^[59]^	IMpassion050 (NCT03726879)	Atezolizumab (PD-L1)	(Atezolizumab + ddAC-PacPH) vs ddAC-PacPH	451	80	No	Yes	III	BC
		(Camrelizumab + chemotherapy) vs chemotherapy								
63	Ren S, et al^[60]^	CameL-Sq (NCT03668496)	Camrelizumab (PD-1)	(Camrelizumab + chemotherapy) vs chemotherapy	298	36	No	Yes	III	Squamous NSCLC
64	Zhou C, et al^[61]^	CameL (NCT03134872)	Camrelizumab (PD-1)	(Camrelizumab + chemotherapy) vs chemotherapy	412	40	No	Yes	III	Non-squamous NSCLC
65	Luo H, et al^[62]^	ESCORT-1st (NCT03691090)	Camrelizumab (PD-1)	(Camrelizumab + chemotherapy) vs chemotherapy	595	22	No	Yes	III	ESCC
66	Yang Y, et al^[63]^	CAPTAIN-1st (NCT03707509)	Camrelizumab (PD-1)	(Camrelizumab + Gemcitabine + Cisplatin) vs (Gemcitabine + Cisplatin)	263	72	No	Yes	III	NC
		(PD-1/PD-L1 + Bevacizumab + chemotherapy) vs (Bevacizumab + chemotherapy)								
67	Socinski MA, et al^[64]^	IMpower150 (NCT02366143)	Atezolizumab (PD-L1)	(Atezolizumab + Bevacizumab + Carboplatin + Paclitaxel) vs (Bevacizumab + Carboplatin + Paclitaxel)	787	72	No	Yes	III	Metastatic non-squamous NSCLC
68	Moore KN, et al^[65]^	IMagyn050/GOG 3015/ENGOT-OV39 (NCT03038100)	Atezolizumab (PD-L1)	(Atezolizumab + CP + Bevacizumab) vs (CP + Bevacizumab)	1286	252	No	Yes	III	Ovarian cancer
69	Sugawara S, et al^[66]^	ONO-4538-52/TASUKI-52 (NCT03117049)	Nivolumab (PD-1)	(Nivolumab + CP + Bevacizumab) vs (CP + Bevacizumab)	548	121	No	Yes	III	NSCLC
		(PD-1/PD-L1 + CTLA-4 + chemotherapy) vs chemotherapy								
70	Paz-Ares L, et al^[67]^	CheckMate 9LA (NCT03215706)	Nivolumab (PD-1)	(Nivolumab + Ipilimumab + chemotherapy) vs chemotherapy	707	78	No	Yes	III	NSCLC
71	Johnson ML, et al A^[35]^	POSEIDON (NCT03164616)	Durvalumab (PD-L1)	(Tremelimumab + Durvalumab + chemotherapy) vs chemotherapy	663	62	No	Yes	III	Metastatic NSCLC
		(PD-1/PD-L1) vs Placebo								
72	Kenmotsu H, et al B^[68]^	IMpower010 (NCT02486718)	Atezolizumab (PD-L1)	Atezolizumab vs Placebo (Non-Japanese)	876	82	No	Yes	III	NSCLC
	Kenmotsu H, et al A^[68]^	IMpower010 (NCT02486718)	Atezolizumab (PD-L1)	Atezolizumab vs Placebo (Japanese)	114	20	No	Yes	III	NSCLC
73	Zhou Q, et al^[69]^	GEMSTONE-301 (NCT03728556)	Sugemalimab (PD-L1)		381	23	No	Yes	III	NSCLC
74	Antonia SJ, et al^[70]^	PACIFIC (NCT02125461)	Durvalumab (PD-L1)	Durvalumab vs Placebo	709	76	Yes	Yes	III	NSCLC
75	Eggermont AMM, et al^[71]^	NCT02362594	Pembrolizumab (PD-1)	Pembrolizumab vs Placebo	1011	136	No	Yes	III	Melanoma
76	Livingstone E, et al A^[72]^	IMMUNED (NCT02523313)	Nivolumab (PD-1)	Nivolumab vs Placebo	107	4	No	Yes	II	Melanoma
77	Long GV, et al^[73]^	KEYNOTE-716 (NCT03553836)	Pembrolizumab (PD-1)	Pembrolizumab vs Placebo	969	133	No	Yes	III	Melanoma
78	Cheng Y, et al^[74]^	ASTRUM-005 (NCT04063163)	Serplulimab (PD-1)	Serplulimab vs Placebo	585	14	No	Yes	III	Extensive SCLC
79	Owonikoko TK, et al C^[75]^	CheckMate 451 (NCT02538666)	Nivolumab (PD-1)	Nivolumab vs Placebo	552	28	Yes	Yes	III	Extensive SCLC
80	Choueiri TK, et al^[76]^	KEYNOTE-564 (NCT03142334)	Pembrolizumab (PD-1)	Pembrolizumab vs Placebo	984	151	No	Yes	III	RCC
81	Kang YK, et al^[77]^	ONO-4538-12, ATTRACTION-2 (NCT02267343)	Nivolumab (PD-1)	Nivolumab vs Placebo	491	24	Yes	Yes	III	GC/GEJC
82	Kelly RJ, et al^[78]^	CheckMate 577 (NCT02743494)	Nivolumab (PD-1)	Nivolumab vs Placebo	792	62	Yes	Yes	III	GC/GEJC
83	Qin S, et al^[79]^	KEYNOTE-394 (NCT03062358)	Pembrolizumab (PD-1)	Pembrolizumab vs Placebo	452	42	Yes	Yes	III	Advanced HC
84	Bajorin DF, et al^[80]^	CheckMate 274 (NCT02632409)	Nivolumab (PD-1)	Nivolumab (Adjuvant) vs placebo	699	72	Yes	Yes	III	UC
85	Bellmunt J, et al^[81]^	IMvigor010 (NCT02450331)	Atezolizumab (PD-L1)	Atezolizumab vs Observation	787	101	No	Yes	III	UC
86	Pal SK, et al^[82]^	IMmotion010 (NCT03024996)	Atezolizumab (PD-L1)	Atezolizumab vs Placebo	783	66	No	Yes	III	RCC
87	Monk BJ, et al B^[51]^	JAVELIN Ovarian 100 (NCT02718417)	Avelumab (PD-L1)	Avelumab vs Observation	662	84	No	Yes	III	Ovarian cancer
88	Luke JJ, et al ^[83]^	KEYNOTE-716 (NCT03553836)	Pembrolizumab (PD-1)	Pembrolizumab vs Placebo	969	104	No	Yes	III	Melanoma
89	Powles T, et al^[84]^	JAVELIN Bladder 100 (NCT02603432)	Avelumab (PD-L1)	Avelumab vs Best Supportive Care (BSC)	689	44	Yes	Yes	III	UC
		(PD-1 + CTLA-4) vs PD-1								
90	Livingstone E, et al B^[72]^	IMMUNED (NCT02523313)	Nivolumab (PD-1)	(Nivolumab + Ipilimumab) vs Nivolumab	111	15	No	Yes	II	Melanoma
91	Weber JS, et al^[85]^	CheckMate 915 (NCT03068455)	Nivolumab (PD-1)	(Nivolumab + Ipilimumab) vs Nivolumab	1833	414	No	Yes	III	Melanoma
92	Larkin J, et al A^[86]^	CheckMate 067 (NCT01844505)	Nivolumab (PD-1)	(Nivolumab + Ipilimumab) vs Nivolumab	626	207	No	Yes	III	Melanoma
93	Hellmann MD, et al A^[7]^	CheckMate 227 (NCT02477826)	Nivolumab (PD-1)	(Nivolumab + Ipilimumab) vs Nivolumab	867	139	No	Yes	III	NSCLC
94	Antonia SJ, et al^[87]^	CheckMate 032 (NCT01928394)	Nivolumab (PD-1)	(Nivolumab + Ipilimumab) vs Nivolumab	152	6	Yes	Yes	I/II	SCLC
		(PD-L1 + chemotherapy) vs PD-L1								
95	Galsky MD, et al A^[23]^	IMvigor130 (NCT02807636)	Atezolizumab (PD-L1)	(Atezolizumab + chemotherapy) vs Atezolizumab	807	75	No	Yes	III	UC
96	Shitara K, et al.2020A^[19]^	KEYNOTE-062 (NCT02494583)	Pembrolizumab (PD-1)	(Pembrolizumab + chemotherapy) vs Pembrolizumab	504	43	No	Yes	III	Gastric cancer
97	Burtness B, et al A^[88]^	KEYNOTE-048 (NCT02358031)	Pembrolizumab (PD-1)	(Pembrolizumab + chemotherapy) vs Pembrolizumab	576	59	No	Yes	III	HNSCC
		(PD-L1 + CTLA-4) vs chemotherapy								
98	de Castro G Jr, et al^[89]^	NEPTUNE (NCT02220894)	Durvalumab (PD-L1)	(Durvalumab + Tremelimumab) vs chemotherapy	809	72	No	Yes	III	NSCLC
99	Hellmann MD, et al B^[7]^	CheckMate 227 (NCT02477826)	Nivolumab (PD-1)	(Nivolumab + Ipilimumab) vs chemotherapy	1146	125	No	Yes	III	NSCLC
100	Doki Y, et al B^[46]^	CheckMate 648 (NCT03143153)	Nivolumab (PD-1)	(Nivolumab + Ipilimumab) vs chemotherapy	626	60	No	Yes	III	Advanced ESCC
101	Baas P, et al^[90]^	CheckMate 743 (NCT02899299)	Nivolumab (PD-1)	(Nivolumab + Ipilimumab) vs chemotherapy	584	58	No	Yes	III	Malignant pleural mesothelioma
102	Shitara K, et al B^[54]^	CheckMate 649 (NCT02872116)	Nivolumab (PD-1)	(Nivolumab + Ipilimumab) vs chemotherapy	792	67	No	Yes	III	Gastro-esophageal cancer
		PD-1/PD-L1 vs CTLA-4								
103	Weber J, et al^[91]^	CheckMate 238 (NCT02388906)	Nivolumab (PD-1)	Nivolumab vs Ipilimumab	905	223	No	Yes	III	Melanoma
104	Larkin J, et al B^[86]^	CheckMate 067 (NCT01844505)	Nivolumab (PD-1)	Nivolumab vs Ipilimumab	624	183	No	Yes	III	Melanoma
105	Schachter J, et al B^[92]^	KEYNOTE-006 (NCT01866319)	Pembrolizumab (PD-1)	Pembrolizumab (2) vs Ipilimumab	534	84	Yes	Yes	III	Melanoma
106	Schachter J, et al C^[92]^	KEYNOTE-006 (NCT01866319)	Pembrolizumab (PD-1)	Pembrolizumab (3) vs Ipilimumab	583	88	Yes	Yes	III	Melanoma
107	Kelley RK, et al C^[93]^	NCT02519348	Durvalumab (PD-L1)	Durvalumab vs Tremelimumab	170	22	Yes	Yes	I/II	Hepatocellular carcinoma
		(PD-1 + CTLA-4) vs Placebo								
108	Motzer RJ, et al^[94]^	CheckMate 914 (NCT03138512)	Nivolumab (PD-1)	(Nivolumab + Ipilimumab) vs Placebo	811	129	No	Yes	III	RCC
109	Livingstone E, et al C^[72]^	IMMUNED (NCT02523313)	Nivolumab (PD-1)	(Nivolumab + Ipilimumab) vs Placebo	106	11	No	Yes	II	Melanoma
		(PD-LI + CTLA-4) vs PD-L1								
110	Powles T, et al A^[95]^	DANUBE (NCT02516241)	Durvalumab (PD-L1)	(Durvalumab + Tremelimumab) vs Durvalumab	685	73	No	Yes	III	UC
111	Kelley RK, et al A^[93]^	NCT02519348	Durvalumab (PD-L1)	(Tremelimumab 300 + durvalumab) vs Durvalumab	175	31	Yes	Yes	I/II	Hepatocellular carcinoma
112	Kelley RK, et al D^[93]^	NCT02519348	Durvalumab (PD-L1)	(Tremelimumab 75 + durvalumab) vs Durvalumab	183	18	Yes	Yes	I/II	Hepatocellular carcinoma

ESCC = esophageal squamous cell carcinoma, GC = gastric cancer, GEJC = gastroesophageal junction cancer, HNSCC = head and neck squamous-cell carcinoma, N/A = not available, NC = nasopharyngeal carcinoma, NSCLC = non-small cell lung cancer, OC = ovarian cancer, RCC = renal-cell carcinoma, RCT = randomized controlled trial, SCLC = small-cell lung cancer, TNBC = triple-negative breast cancer, UC = urothelial carcinoma.

Among all the selected clinical trials, phase III clinical trials accounted for the highest proportion (n = 89),^[1–16,18–24,26–30,32–71,73–86,88–92,94,95]^ followed by phase II clinical trials (n = 7; Table 1).^[4,17,25,31,72,87,93]^ non-small cell lung cancer (NSCLC) was still the most reported tumor type (n = 32; Table 1).^[1,3,4,6,7,9,11,16,20,21,24–36,60,61,64,66–70,89]^ Previous therapies were found in 26 clinical trials (n = 25; Table 1),^[1,3–6,8–10,12,13,15,17,18,20,36,70,75,77–80,84,87,92,93]^ whereas PD-1 or PD-L1 inhibitors were prescribed as the first-line treatment choice in other studies (n = 70).^[2,7,11,14,16,19,21–35,37–69,71–74,76,81–83,85,86,88–91,94,95]^ Sixty-six clinical trials were found to be PD-1 related,^[1–5,7,9–14,16–20,24–32,43–50,52–58,60–63,66,67,71–80,83,85–88,90–92,94]^ while the rest were PD-L1 related.^[6,8,15,21–23,33–42,51,59,64,65,68–70,81,82,84,89,93,95]^

No fewer than 3 sets of data were required for the final analysis (Table 1). Based on the composition of PD-1/PD-L1 related treatment regimens, all the data were divided into 12 groups for meta-analyses and listed as follows: Group 1 (PD-1/PD-L1 vs chemotherapy),^[1–23]^ Group 2 ((PD-1/PD-L1 + chemotherapy) vs chemotherapy)^[24–59]^; Group 3 ((Camrelizumab + chemotherapy) vs chemotherapy)^[60–63]^; Group 4 ((PD-1/PD-L1 + Bevacizumab + chemotherapy) vs (Bevacizumab + chemotherapy))^[55,64–66]^; Group 5 ((PD-1/PD-L1 + CTLA-4 + chemotherapy) vs chemotherapy)^[35,37,67]^; Group 6 (PD-1/PD-L1 vs placebo)^[68–84]^; Group 7 ((PD-1 + CTLA-4) vs PD-1)^[7,72,78,85–87]^; Group 8 ((PD-L1 + chemotherapy) vs PD-L1)^[15,19,23,88]^; Group 9 ((PD-L1 + CTLA-4) vs chemotherapy)^[7,46,54,89,90]^; Group 10 (PD-1/PD-L1 vs CTLA-4)^[9,91–93]^; Group 11 ((PD-1 + CTLA-4) vs placebo)^[72,75,94]^; Group 12 ((PD-L1 + CTLA-4) vs PD-L1).^[93,95]^

### 3.2. Risk of bias

Assessment of bias for all enrolled clinical trials, including selection bias, performance bias, detection bias, attrition bias, reporting bias, and other biases, were completed and are displayed in (Figure S1, Supplemental Digital Content 1).^[1–95]^ No high-risk biases were observed.

### 3.3. Risk of rash for all grades in Group 1 (PD-1/PD-L1 vs chemotherapy)

**Figure 2.**
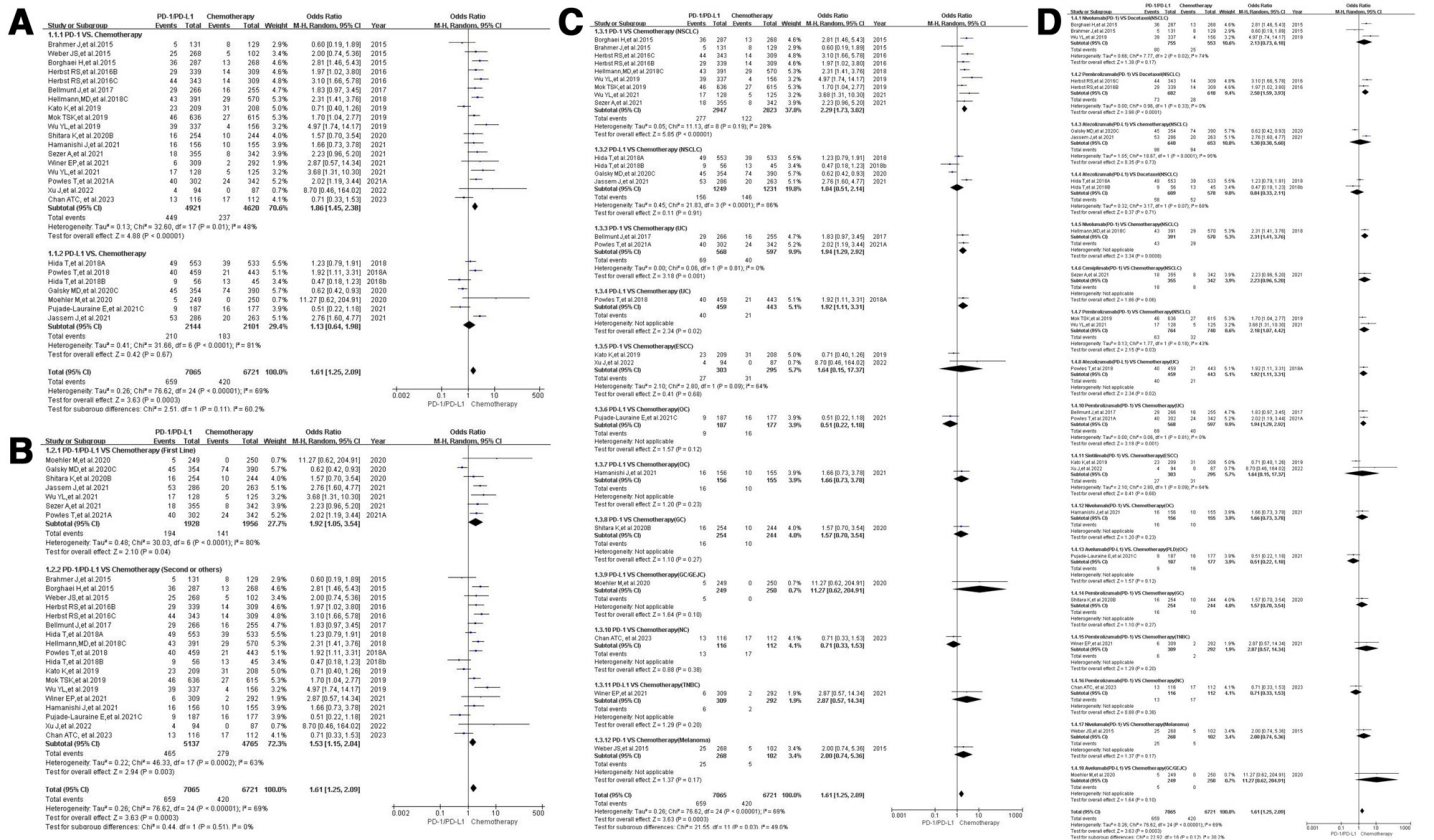
Forest plots of the analysis results in Group 1 (PD-1/PD-L1 vs chemotherapy). (A) Analysis results of the odds ratio (OR) for rash occurrence calculated using the random effects (RE) model: Subgroup analyses were mainly based on the type of immune drug (PD-1 or PD-L1). (B) Analysis results of the OR for rash occurrence calculated using the RE model: subgroup analyses were mainly based on the treatment lines (first or second line). (C) Analysis results of the OR for rash occurrence calculated using the RE model: subgroup analyses were mainly based on both immune drug and tumor types. (D) Analysis results of the OR for rash occurrence calculated using the RE model: subgroup analyses were mainly based on both immune drug names and tumor types. PD-1 = programmed cell death-1, PD-L1 = programmed cell death ligand 1.

A total of 23 clinical trials were selected for this group.^[1–23]^ Compared with chemotherapy, PD-1/PD-L1 increased the risk of rash occurrence (OR = 1.61, 95% CI: [1.25–2.01]; *I*^2^ = 69%, *Z* = 3.63, *P* = .0002; Fig. 2). Moreover, this risk appeared to be more pronounced in the PD-1 subgroup (OR = 1.86, 95% CI: [1.45–2.38]; *I*^2^ = 48%, *Z* = 4.88, *P* < .00001; Fig. 2A).^[1–5,7,9–14,16–20]^ When PD-1/PD-L1 was prescribed as the first-line treatment option, a higher risk trend was observed (OR = 1.92, 95% CI: [1.05–3.54]; *I*^2^ = 80%, *Z* = 2.10, *P* = .04; Fig. 2B).^[2,7,11,14–16,19,21–23]^

**Figure 3.**
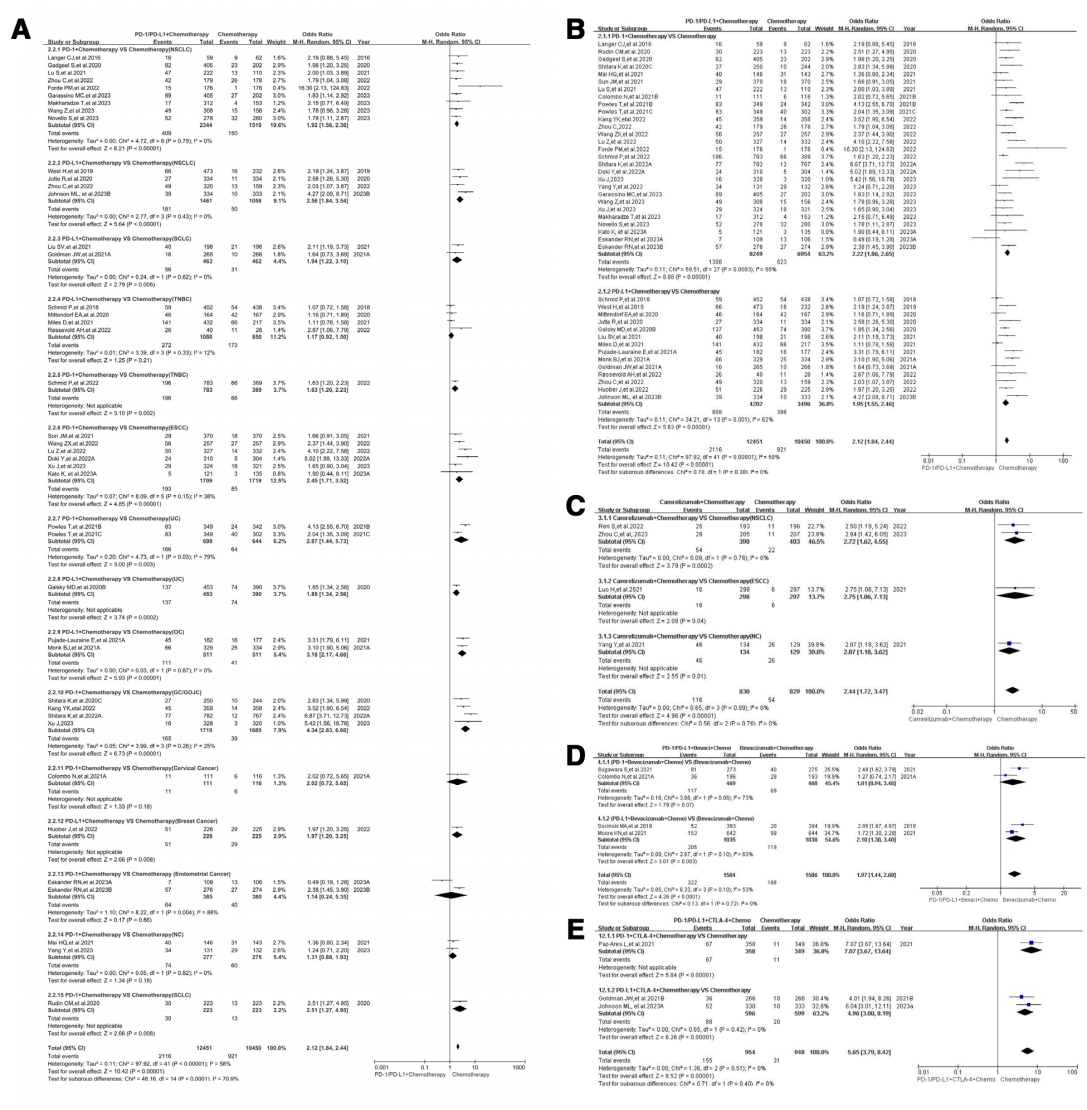
Forest plots of the analysis results in Group 2, Group 3, Group 4, and Group 5. (A) Analysis results of the odds ratio (OR) for rash occurrence calculated using the random effects (RE) model in Group 2: subgroup analyses were mainly based on both immune drug and tumor types. (B) Analysis results of the OR for rash occurrence calculated by the RE model in Group 2; subgroup analyses were mainly based on the type of immune drug (PD-1 or PD-L1). (C) Analysis results of the OR for rash occurrence calculated by the RE model in Group 3; subgroup analyses were mainly based on tumor types. (D) Analysis results of the OR for rash occurrence calculated by the RE model in Group 4; subgroup analyses were mainly based on the type of immune drug (PD-1 or PD-L1). (E) Analysis results of the OR for rash occurrence calculated by the RE model in Group 5; subgroup analyses were mainly based on the type of immune drug (PD-1 or PD-L1). PD-1 = programmed cell death-1, PD-L1 = programmed cell death ligand 1.

When subgroup analyses were performed according to tumor type, the risk of rash occurrence varied significantly among different tumor types (df = 11 (*P* = .03); Fig. 2C). Subgroup analysis suggested that PD-1 might pose a higher risk of rash occurrence in NSCLC (OR = 2.29, 95% CI: [1.73–3.02]; *I*^2^ = 28%, *Z* = 5.85, *P* < .00001; Fig. 3C), and similar higher risk trends were observed in the urothelial carcinoma (UC) subgroup (OR = 1.94). However, the inclusion of no fewer than 3 clinical trials was only found in the NSCLC subgroup. Therefore, further elucidation of the differences in the risk of specific tumor types is still controversial, and more reports from relevant clinical trials are needed (Fig. 2C).

Further subgroup analysis suggested that high heterogeneity (*I*^2^ = 57%; Fig. 2) might be associated with the 2 clinical trials related to NSCLC.^[21,23]^ After further checking the original data, it was inferred that heterogeneity might originate mainly from the data itself (Fig. 2D). The corresponding funnel plots are shown in (Figure S2, Supplemental Digital Content 2).

### 3.4. Risk of rash for all grades in Group 2, Group 3, Group 4, and Group 5

Forty clinical trials were selected for Group 2.^[14,15,19,23–59]^ Through analysis, it was found that combination with chemotherapy increased the risk of rash occurrence (OR = 2.12, 95% CI:[1.84–2.44]; *I*^2^ = 58%, *Z* = 10.42, *P* < .00001; Fig. 3A, B). The subgroup analysis also suggested that the PD-1 subgroup had a slightly higher risk trend (OR = 2.22, 95% CI: [1.86–2.65]; *I*^2^ = 55%, *Z* = 8.88, *P* < .00001; Fig. 3B). Further subgroup analysis indicated that the risk of rash occurrence was significantly higher in the GC/GOJC subgroup (OR = 4.34, 95% CI: [2.83–6.66]; *I*^2^ = 25%, *Z* = 6.73, *P* < .00001; Fig. 3A),^[19,52–54]^ when PD-1 combined with chemotherapy was compared with chemotherapy alone. High heterogeneity (*I*^2^ = 58%; Fig. 3A, B) was found, and subgroup analysis suggested that it might be mainly caused by the clinical trial related to UC (*I*^2^ = 79%; Fig. 3A).^[14]^ The corresponding funnel plots are provided in (Figure S3A, B, Supplemental Digital Content 3).

Four clinical trials were selected in Group 3.^[60–63]^ Similarly, Camrelizumab in combination with chemotherapy increased the risk of rash (OR = 2.44, 95% CI:[1.72–3.47]; *I*^2^ = 0%, *Z* = 4.96, *P* < .00001; Fig. 3C). When PD-1 or PD-L1 inhibitors were combined with other drugs (Group 4, Group 5),^[35,37,55,64–67]^ similar risk trends were observed (Fig. 3D, E), especially for CTLA-4 (OR = 5.65, 95% CI: [3.79–8.42]; *I*^2^ = 0%, *Z* = 8.52, *P* < .00001; Fig. 3E).^[35,37,67]^ The corresponding funnel plots are provided in (Figure S3C, E, Supplemental Digital Content 3).

### 3.5. Risk of rash for all grades in Group 6 (PD-1/PD-L1 vs placebo)

**Figure 4.**
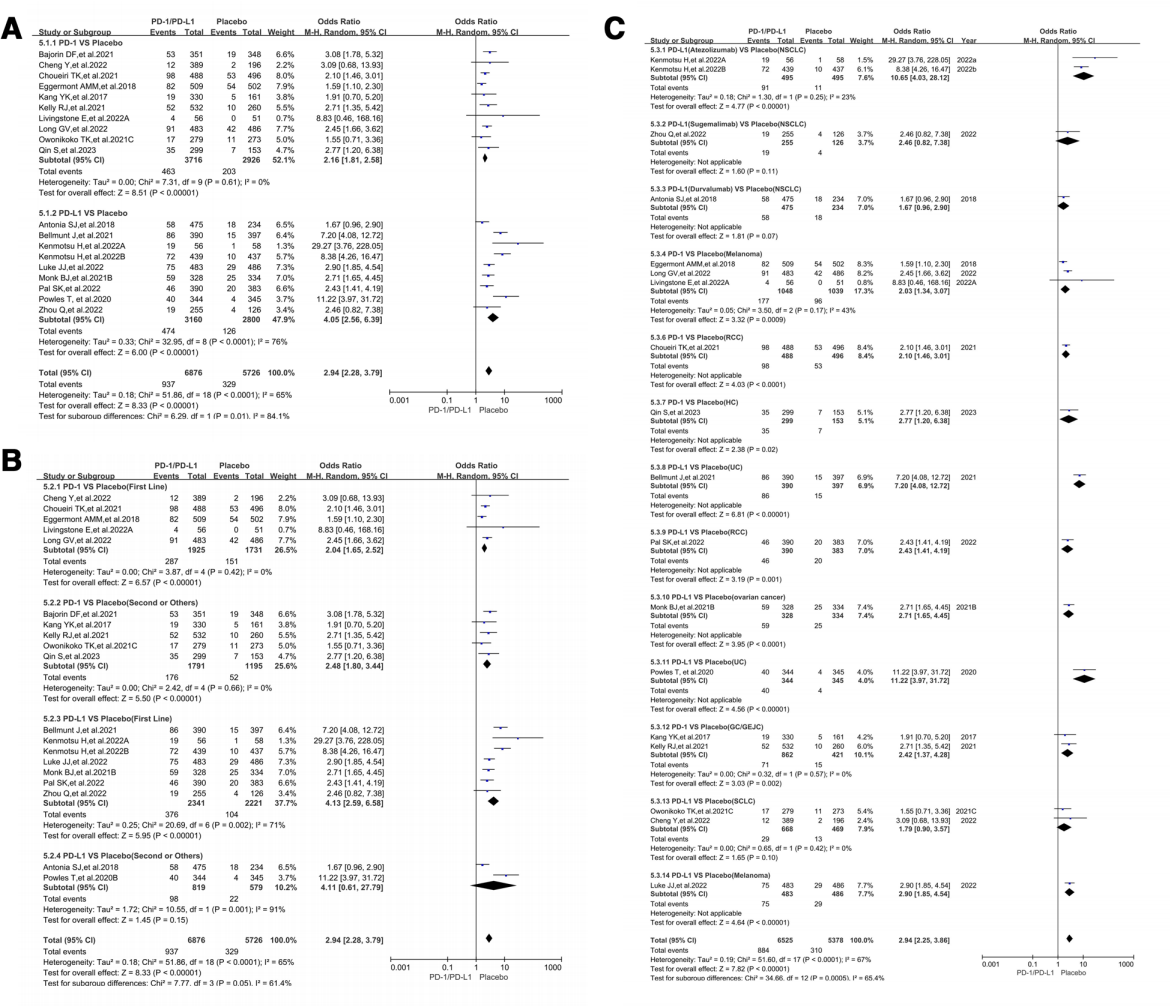
Forest plots of the analysis results in Group 6 (PD-1/PD-L1 vs placebo). (A) Analysis results of the odds ratio (OR) for rash occurrence calculated using the random effects (RE) model: subgroup analyses were mainly based on the type of immune drug (PD-1 or PD-L1). (B) Analysis results of the OR for rash occurrence calculated using the RE model: subgroup analyses were mainly based on the treatment lines (first or second line). (C) Analysis results of the OR for rash occurrence calculated using the RE model: subgroup analyses were mainly based on both immune drug and tumor types. PD-1 = programmed cell death-1, PD-L1 = programmed cell death ligand 1.

Eighteen clinical trials were selected from Group 6.^[51,68–84]^ Compared to placebo, the use of PD-1 or PD-L1 inhibitors significantly increased the risk of rash occurrence (OR = 2.94, 95% CI: [2.28–3.79]; *I*^2^ = 65%, *Z* = 8.33, *P* < .00001; Fig. 4).^[51,68–84]^ In contrast to the above findings (Fig. 2A), subgroup analysis suggested a higher risk of rash occurrence in the PD-L1 subgroup (OR = 4.05, 95% CI: [2.56–6.39]; *I*^2^ = 76%, *Z* = 6.00, *P* < .00001; Fig. 4A).^[51,68–70,81–84]^ A similar risk trend was also observed in further analyses (Fig. 4B). Subgroup analysis indicated that high heterogeneity (*I*^2^ = 65%) might be related to the clinical trials on melanoma (*I*^2^ = 43%, Fig. 4C).^[71–73]^ All the corresponding funnel plots are shown in (Figure S4, Supplemental Digital Content 4).

### 3.6. Risk of rash for all grades in Group 7, Group 8, Group 9, Group 10, Group 11, and Group 12

**Figure 5.**
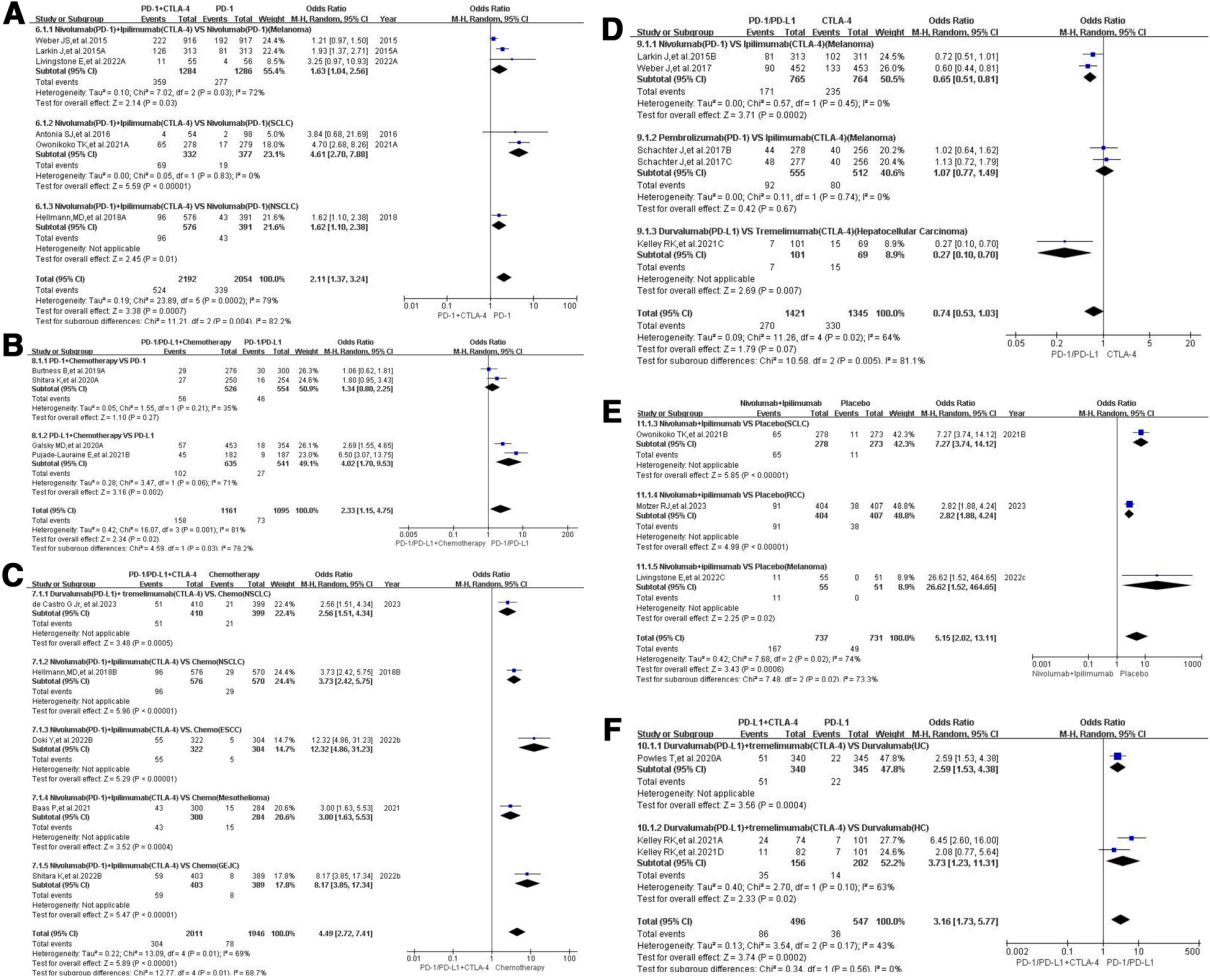
Forest plots of the analysis results in Group 7, Group 8, Group 9, Group 10, Group 11, and Group 12. (A) Analysis results of the odds ratio (OR) for rash occurrence calculated using the random effects (RE) model in Group 7 (PD-1 + CTLA-4 vs PD-1): Subgroup analyses were mainly based on tumor type. (B) Analysis results of the OR for rash occurrence calculated using the RE model in Group 8 (PD-1/PD-L1 + chemotherapy vs PD-1/PD-L1): subgroup analyses were mainly based on the type of immune drug (PD-1 or PD-L1). (C) Analysis results of the OR for rash occurrence calculated using the RE model in Group 9 (PD-1/PD-L1 + CTLA-4 vs chemotherapy): subgroup analyses were mainly based on both immune drug and tumor types. (D) Analysis results of the OR for rash occurrence calculated by the RE model in Group 10 (PD-1/PD-L1 vs CTLA-4): subgroup analyses were mainly based on both immune drug names and tumor types. (E) Analysis results of the OR for rash occurrence calculated using the RE model in Group 11 (PD-1 + CTLA-4 vs placebo): subgroup analyses were mainly based on tumor types. (F) Analysis results of the OR for rash occurrence calculated using the RE model in Group 12 (PD-L1 + CTLA-4 vs PD-L1): subgroup analyses were mainly based on tumor types. PD-1 = programmed cell death-1, PD-L1 = programmed cell death ligand 1.

Six clinical trials were selected in Group 7 (PD-1 + CTLA-4 vs PD-1).^[7,72,75,85–87]^ Analysis revealed that adding CTLA-4 to PD-1 significantly increased the risk of rash occurrence (OR = 2.11, 95% CI: [1.37–3.24]; *I*^2^ = 79%, *Z* = 3.38, *P* = .0007; Fig. 5A).^[7,72,75,85–87]^ Subgroup analysis indicated that high heterogeneity (*I*^2^ = 79%) might be mainly related to the clinical trials on melanoma (*I*^2^ = 72%, Fig. 5A).^[72,85,86]^ The corresponding funnel plots are presented in (Figure S5A, Supplemental Digital Content 5).

Four clinical trials were selected in Group 8 (PD-L1 + chemotherapy vs PD-L1).^[15,19,23,88]^ It was also found that adding chemotherapy to PD-L1 significantly increased the risk of rash occurrence (OR = 2.33, 95% CI: [1.15–4.75]; *I*^2^ = 81%, *Z* = 2.34, *P* = .02; Fig. 5B).^[15,19,23,88]^ Subgroup analysis indicated that high heterogeneity (*I*^2^ = 81%) might be related to the 2 clinical trials (*I*^2^ = 71%, Fig. 5B).^[15,23]^ The corresponding funnel plots are presented in (Figure S5B, Supplemental Digital Content 5).

Five clinical trials were selected in Group 9 (PD-L1 + CTLA-4 vs chemotherapy).^[7,46,54,89,90]^ Through analysis, it was also found that adding CTLA-4 to PD-L1 significantly increased the risk of rash occurrence (OR = 4.49, 95% CI: [2.72–7.41]; *I*^2^ = 69%, *Z* = 5.89, *P* < .00001; Fig. 5C)^[7,46,54,89,90]^ compared with chemotherapy. Subgroup analysis indicated that the high heterogeneity (*I*^2^ = 69%) might be related to the data. The corresponding funnel plots are presented in (Figure S5C, Supplemental Digital Content 5).

Four clinical trials were selected for Group 10 (PD-1/PD-L1 vs CTLA-4).^[86,91–93]^ Through analysis, it was also found that the risk of rash occurrence caused by PD-1/PD-L1 was lower than that of CTLA-4 (OR = 0.74, 95% CI: [0.53–1.03]; *I*^2^ = 64%, *Z* = 1.79, *P* = .07; Fig. 5D).^[86,91–93]^ Subgroup analysis indicated that the high heterogeneity (*I*^2^ = 64%) might be related to the data. The corresponding funnel plots are presented in (Figure S5D, Supplemental Digital Content 5).

Three clinical trials were selected in Group 11 (PD-1 + CTLA-4 vs placebo).^[72,75,94]^ Compared to placebo, PD-1 plus CTLA-4 significantly increased the risk of rash occurrence (OR = 5.15, 95% CI: [2.02–13.11]; *I*^2^ = 74%, *Z* = 3.43, *P* = .0006; Fig. 5E).^[72,75,94]^ Subgroup analysis indicated that the high heterogeneity (*I*^2^ = 74%) might be related to the data. The corresponding funnel plots are presented in (Figure S5E, Supplemental Digital Content 5).

Two clinical trials were selected for Group 12 (PD-LI + CTLA-4 vs PD-L1).^[93,95]^ It was also found that adding CTLA-4 to PD-L1 significantly increased the risk of rash occurrence (OR = 3.16, 95% CI: [1.73–5.77]; *I*^2^ = 43%, *Z* = 3.74, *P* = .0002; Fig. 5F).^[93,95]^ The subgroup analysis indicated that moderate heterogeneity (*I*^2^ = 43%) might be related to the data. The corresponding funnel plots are shown in (Figure S5F, Supplemental Digital Content 5).

## 4. Discussion

Rash is one of the most common toxic skin reactions associated with PD-1/PD-L1.^[1–95]^ Although it rarely affects patients’ treatment plans, it can have a serious impact on their quality of life. Therefore, it has been increasingly valued in clinical work.^[1–95]^ Identifying the risk factors for rashes in complex treatment regimens is a major dilemma faced by clinicians. With the recent update of a large amount of clinical trial data related to PD-1/PD-L1 inhibitors, we have realized the need to update our previous analysis results to better evaluate the risk of PD-1/PD-L1 related rash.^[96]^

After strict screening, 95 clinical trials were selected and used for the final comprehensive analysis (Fig. 1; Table 1).^[1–95]^ A comprehensive quality evaluation was conducted on all data, and no high-risk bias-related factors were found (Figure S1, Supplemental Digital Content 1), which further laid the foundation for the authenticity and reliability of the analysis results. According to different treatment regimens, all clinical trials were divided into 12 groups (Table 1). To further increase the reliability of the analysis results, it was stipulated that all groups should include at least 3 or more sets of data, which was also the key point for improvement in the updated analysis.^[96]^

Regardless of whether PD-1/PD-L1 was compared with chemotherapy or placebo (Figs. 2 and 4),^[14,15,19,23–59,68–70,81–84]^ the analysis suggested that both PD-1 and PD-L1 increased the risk of rash occurrence. When the control groups were different, the risk trends of PD-1 versus PD-L1-induced rashes also differed (Fig. 2A and Fig. 4A).^[14,15,19,23–59,68–70,81–84]^ Due to the lack of clinical trials on the head-to-head comparison between PD-1 and PD-L1, further research is needed to determine the differences in rash risk between PD-1 and PD-L1 inhibitors.

In the combination therapy regimen based on PD-1/PD-L1 (Fig. 3, Fig. 5A–C, Fig. 5E, F), it was found that the risk of rash occurrence increased in combination with chemotherapy, CTLA-4, bevacizumab, or others. Furthermore, in combination with immunosuppressive drugs, the increased risk of this occurrence was particularly significant (Figs. 3E, 5C, E, F). In other words, the risk of rash occurrence caused by immunosuppressive antitumor drugs might be higher than that of other antitumor drugs, which would be helpful for guiding the option of drug prescription and discontinuation in clinical studies.

Through direct and indirect comparison, it was found that the risk of rash caused by PD-1/PD-L1 inhibitors was significantly lower than that of CTLA-4 drugs (Fig. 5D), and this risk was significantly increased when the 2 immunosuppressants were used in combination (Fig. 5A–C, E, F). This reminded us to be particularly vigilant about the risk of rash occurrence when 2 immunosuppressive drugs were used in combination in clinical work. The analysis results were significantly more comprehensive and systematic than before,^[96]^ which further increased the reliability and authority of the conclusion.

Throughout the analysis process, varying degrees of heterogeneity were observed, and corresponding subgroup analyses were conducted to infer the sources of heterogeneity (Figs. 2–5). After verifying the original data, we found that heterogeneity was more likely to be caused by the data. Therefore, we believe that heterogeneity had little impact on the results. No significant publication bias was found when evaluating the corresponding funnel plots (Figures S2–S5, Supplemental Digital Content 4); no significant publication bias was found. The above results further enhance the authenticity and reliability of our results.

In summary, PD-1 or PD-L1 tends to increase or exacerbate the risk of rash. Informing patients of the potential rash risk in advance and helping them psychologically prepare will help improve their treatment compliance and enhance clinicians’ control over the entire treatment process. Moreover, for patients with underlying skin diseases, it may be necessary to provide them with corresponding skin disease-related warning monitoring in advance. If the patient’s immunotherapy is effective and the rash is tolerable, continuing immunotherapy after symptomatic treatment may be a better option.

## 5. Conclusions

Both PD-1 and PD-L1 increase the risk of rash occurrence, especially when used in combination with other immunosuppressive antitumor drugs. These findings will help guide clinicians in determining the source of rashes in clinical practice and further guide medication selection.

## 6. Limitations

Despite our utmost efforts to incorporate the results of clinical trials with as many samples as possible, potential publication bias and underreporting of adverse events may still exist, which is the main limitation of this study. In addition, although some literature was searched, relevant data could not be obtained, which is another limitation of the study.

## Acknowledgments

The corresponding author (Y.T.) designed and drafted the manuscript. Q.J., J.L., Z.L., C.Z., and Q.D. were appointed for literature screening, and all data analyses were completed by Y.T., who received the final version of the manuscript and made the corresponding corrections. The corresponding author was responsible for coordinating all disagreements and making decisions regarding submission of the manuscript.

## Author contributions

**Data curation:** Junru Liu, Chi Zhang.

**Formal analysis:** Qunqun Jiang, Yuan Tian.

**Methodology:** Qunqun Jiang, Chi Zhang, Yuan Tian.

**Project administration:** Yuan Tian.

**Software:** Junru Liu, Zhuoqi Li, Chi Zhang.

**Supervision:** Junru Liu, Zhuoqi Li, Chi Zhang, Qi Dang.

**Validation:** Junru Liu, Zhuoqi Li, Chi Zhang, Qi Dang.

**Visualization:** Zhuoqi Li.

**Writing – original draft:** Qunqun Jiang.

**Writing – review & editing:** Junru Liu, Qi Dang, Yuan Tian.










