# Supplementary figures and images for: The risk of rash for cancer patients treated with PD-1/PD-L1 inhibitors: An updated systematic review and meta-analysis
Source: Medicine (Baltimore). 2026 Jul 10;105(28):e49720. doi: 10.1097/MD.0000000000049720 (PMC13363018; doi:10.1097/MD.0000000000049720)

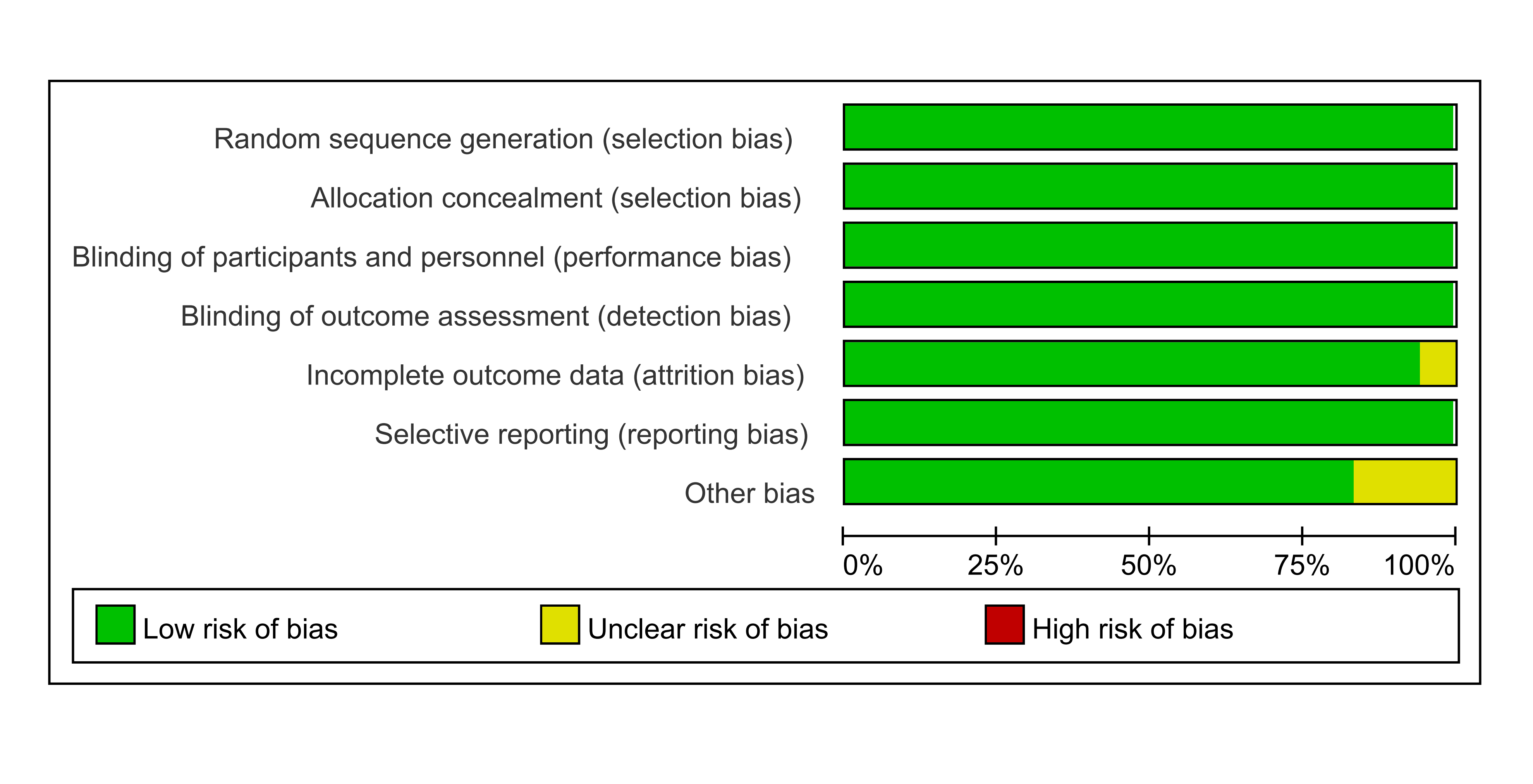

Supplement: Supplementary file 1 [file medi-105-e49720-s001.tif]

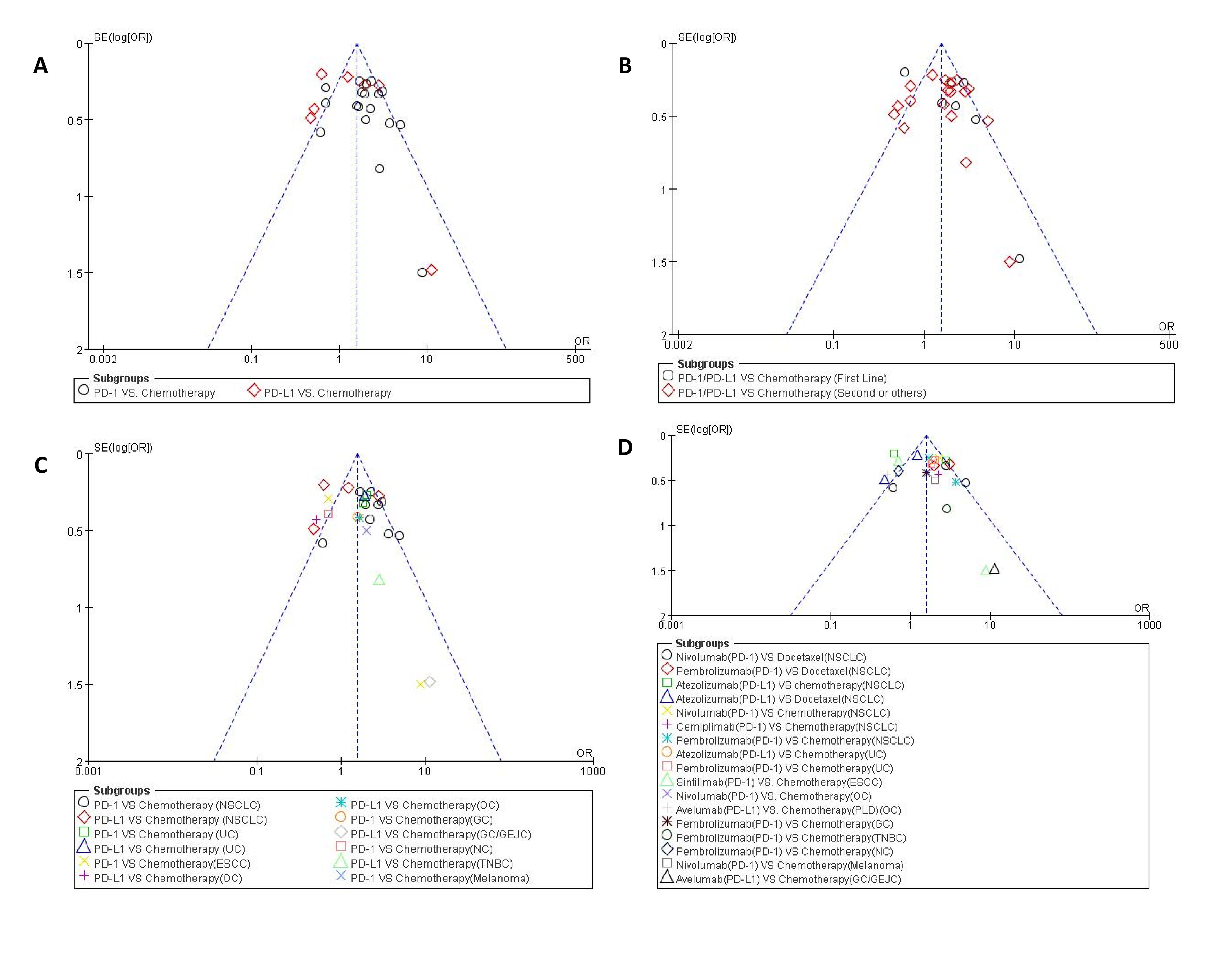

Supplement: Supplementary file 2 [file medi-105-e49720-s002.tif]

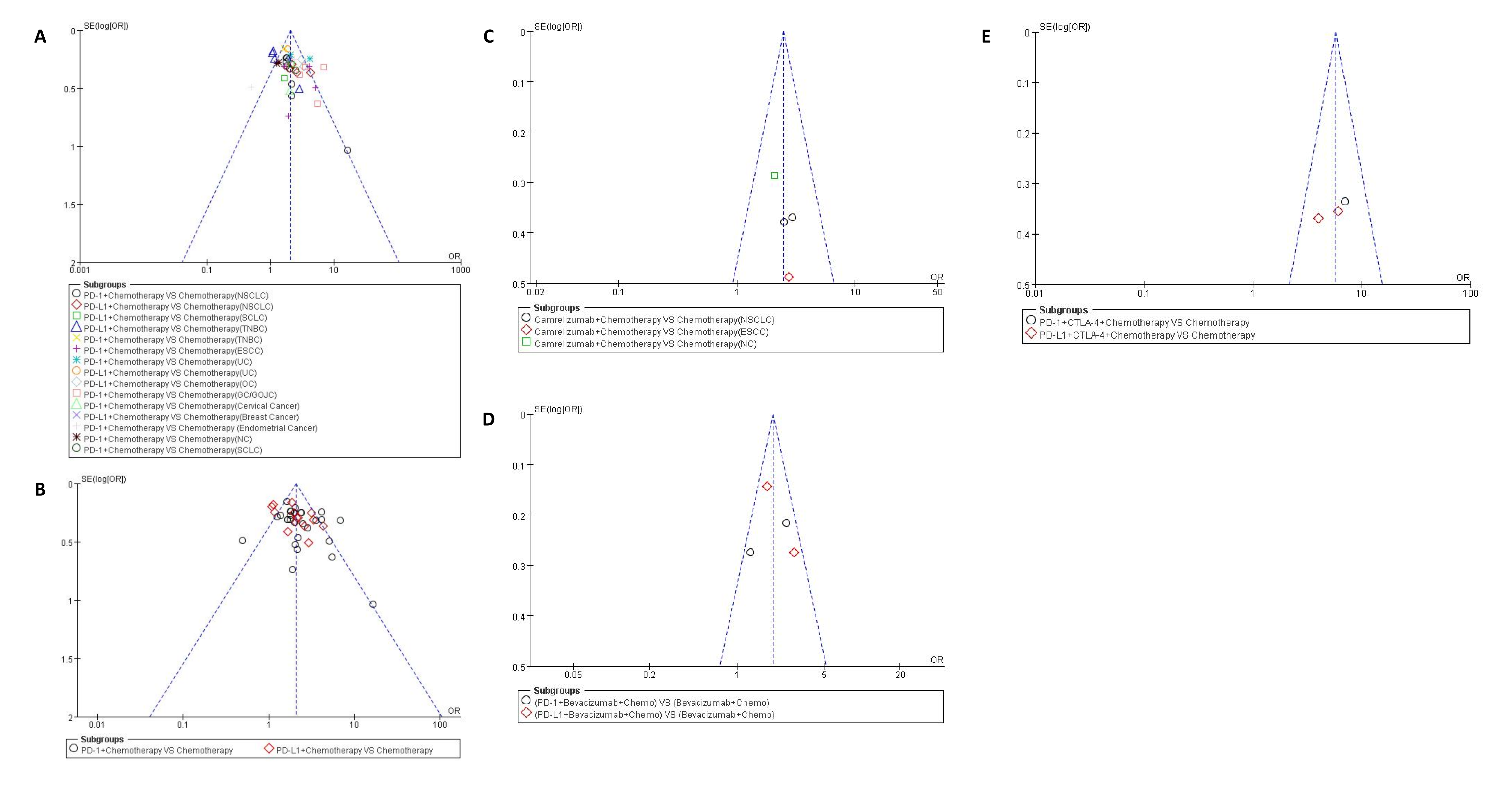

Supplement: Supplementary file 3 [file medi-105-e49720-s003.tif]

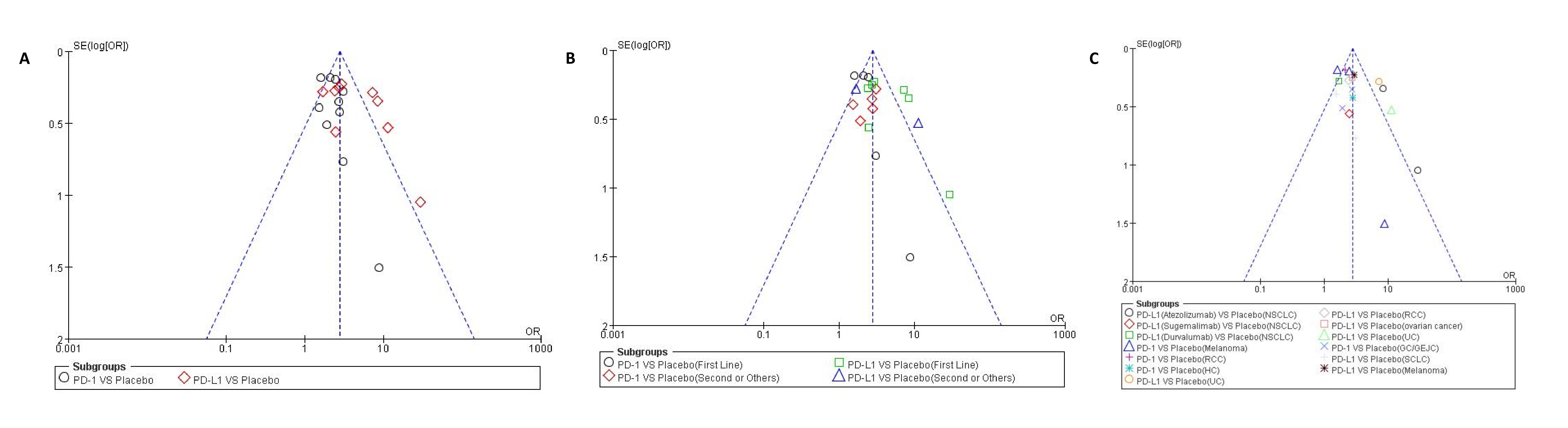

Supplement: Supplementary file 4 [file medi-105-e49720-s004.tif]

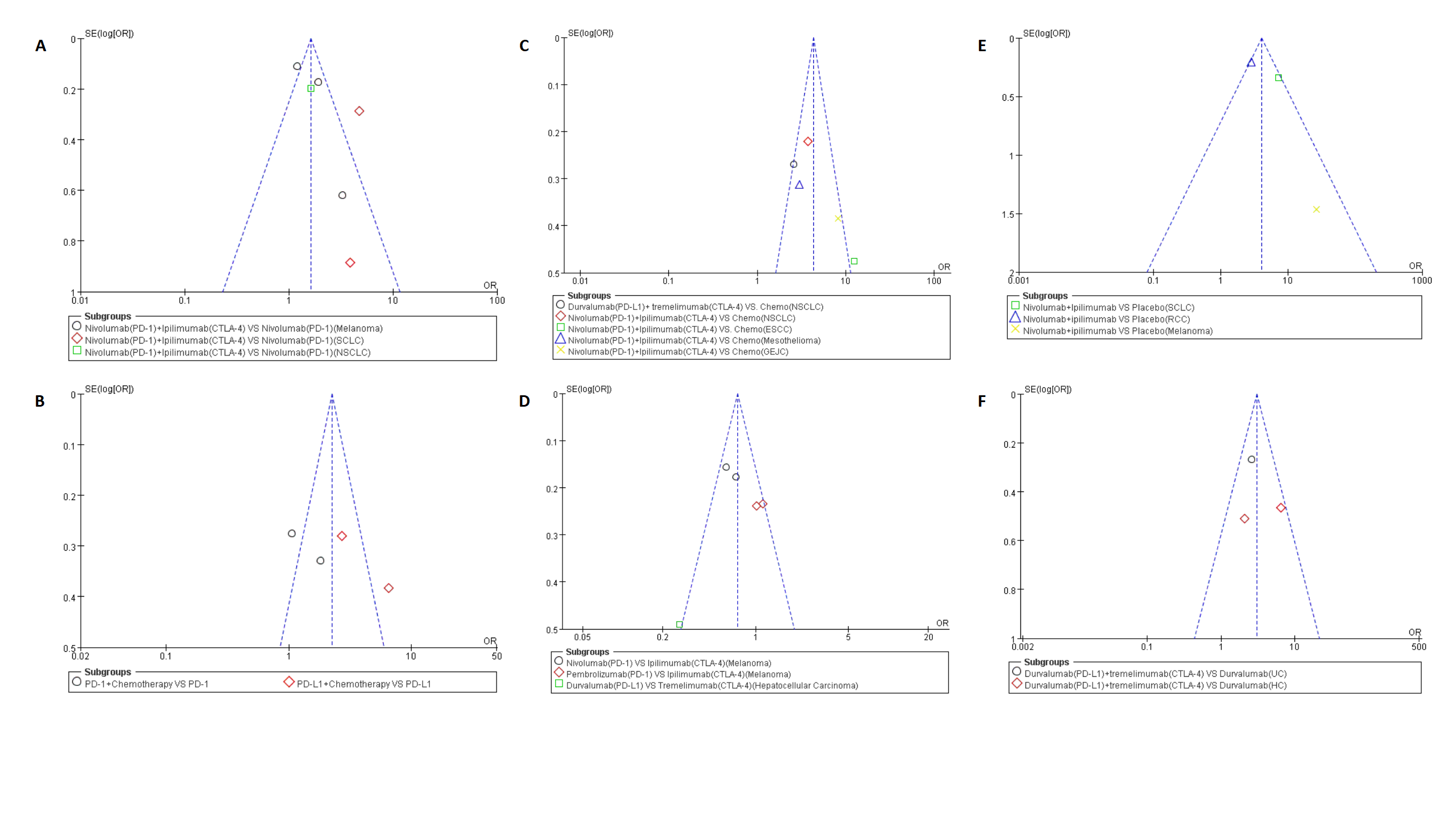

Supplement: Supplementary file 5 [file medi-105-e49720-s005.tif]
